# Differential survival following trastuzumab treatment based on quantitative HER2 expression and HER2 homodimers in a clinic-based cohort of patients with metastatic breast cancer

**DOI:** 10.1186/1471-2407-10-56

**Published:** 2010-02-23

**Authors:** Masakazu Toi, Jeff Sperinde, Weidong Huang, Shigehira Saji, John Winslow, Xueguang Jin, Yuping Tan, Shinji Ohno, Seigo Nakamura, Hiroji Iwata, Norikazu Masuda, Kenjiro Aogi, Satoshi Morita, Christos Petropoulos, Michael Bates

**Affiliations:** 1 Faculty of Medicine, Kyoto University, Kyoto, Japan; 2 Department of Research and Development, Monogram Biosciences, Inc., South San Francisco, CA, USA; 3 Department of Clinical Research, Monogram Biosciences, Inc, South San Francisco, CA, USA; 4 Division of Clinical Trials and Research and Department of Surgery, Tokyo Metropolitan Cancer and Infectious Disease Center, Komagome Hospital, Tokyo, Japan; 5 Division of Breast Oncology, National Kyushu Cancer Center, Fukuoka, Japan; 6 Breast Surgical Oncology, St Luke's International Hospital, Tokyo, Japan; 7 Department of Breast Oncology, Aichi Cancer Center Hospital, Aichi, Japan; 8 Department of Surgery, National Hospital Organization, Osaka National Hospital, Osaka, Japan; 9 Department of Breast Oncology, National Hospital Organization, Shikoku Cancer Center, Matsuyama, Japan; 10 Department of Biostatistics and Epidemiology, Yokohama City University Medical Center, Yokohama, Japan

## Abstract

**Background:**

We have recently described the correlation between quantitative measures of HER2 expression or HER2 homodimers by the HERmark assay and objective response (RR), time-to progression (TTP), and overall survival (OS) in an expanded access cohort of trastuzumab-treated HER2-positive patients with metastatic breast cancer (MBC) who were stringently selected by fluorescence in situ hybridization (FISH). Multivariate analyses suggested a continuum of HER2 expression that correlated with outcome following trastuzumab. Here we investigate the relationship between HER2 expression or HER2 homodimers and OS in a clinic-based population of patients with MBC selected primarily by IHC.

**Methods:**

HERmark, a proximity-based assay designed to detect and quantitate protein expression and dimerization in formalin-fixed paraffin-embedded (FFPE) tissues, was used to measure HER2 expression and HER2 homodimers in FFPE samples from patients with MBC. Assay results were correlated with OS using univariate Kaplan-Meier, hazard function plots, and multivariate Cox regression analyses.

**Results:**

Initial analyses revealed a parabolic relationship between continuous measures of HER2 expression and risk of death, suggesting that the assumption of linearity for the HER2 expression measurements may be inappropriate in subsequent multivariate analyses. Cox regression analyses using the categorized variable of HER2 expression level demonstrated that higher HER2 levels predicted better survival outcomes following trastuzumab treatment in the high HER2-expressing group.

**Conclusions:**

These data suggest that the quantitative amount of HER2 expression measured by Hermark may be a new useful marker to identify a more relevant target population for trastuzumab treatment in patients with MBC.

## Background

Over-expression of HER2 has been linked to both adverse prognosis and improved responsiveness to treatment with trastuzumab (Herceptin, Genentech) in a sub-population of metastatic breast cancers [1-5]. Trastuzumab offers significant disease-free and overall survival advantages in both the metastatic and adjuvant settings in patients with HER2 over-expressing tumors [6-11]. Currently, the selection of patients with breast cancer for treatment with trastuzumab is based on the measurement of HER2 receptor protein expression by immunohistochemistry (IHC), or by the presence of HER2 gene amplification as detected by FISH. Despite the success of trastuzumab, recent data from NCCTG 9831, NSABP B-31, and CALGB 150002 have called into question the accuracy of the current methods used to identify those patients who are most likely to benefit from treatment with trastuzumab [12-14]. New approaches to the accurate quantitation of HER2 expression and HER2 dimerization in FFPE breast tumor specimens may have the potential to identify responders more precisely.

We have previously reported that quantitative measures of HER2 expression or HER2 homodimers in FFPE specimens from MBC patients enrolled in a trastuzumab expanded access program identified sub-populations of patients with different clinical outcomes, such that patients whose tumors expressed higher levels of HER2 or HER2 homodimers lived longer [15]. Those patients had been stringently selected for trastuzumab treatment primarily by FISH (90%). Here we describe the correlation of quantitative measurements of HER2 expression (H2T) or HER2 homodimers (H2D) with OS following trastuzumab exposure in a cohort of patients with MBC who were selected for treatment by IHC (88%) or FISH (12%) in a routine clinic setting. Quantitative HER2 measurements were made by the HERmark assay (Monogram Biosciences) using FFPE breast tumor specimens. HER2 homodimers are defined as HER2:HER2 associations that are sufficient to lead to HER2 phosphorylation and subsequent activation of downstream signaling pathways. These could include HER2:HER2 dimers or HER2 monomers that are in close proximity (< 80 nm) as a result of pathological over-expression.

## Methods

### The VeraTag technology

The VeraTag technology is a proximity-based method designed to accurately and reproducibly quantitate protein expression and protein-protein complexes, including cell surface-expressed protein dimers, in FFPE specimens. The first assays developed for clinical testing using the VeraTag platform measure HER2 protein expression (H2T) and HER2 homodimer (H2D) levels accurately and reproducibly, and are referred to as HERmark. These assays have undergone a formal validation at Monogram Biosciences and are regulated by the College of American Pathologists under the specifications established by CLIA (Clinical Laboratory Improvement Amendment) [16]. IHC (Herceptest, DAKO) and FISH assays were performed according to standard protocols at the local institutions where the patients were treated.

### Description of the clinical cohort

This cohort is composed of 75 patients drawn from six oncology clinics in Japan. Patients were included if they had metastatic breast cancer, had been treated with at least one chemotherapy regimen prior to receiving trastuzumab either as single agent or in combination with chemotherapy, and had FFPE specimens available for testing by the HERmark assay. Assessments of HER2 over-expression by IHC or FISH were performed by the pathologist at the local hospital. All clinical follow-up was performed at the local hospital, and clinical outcome data were drawn from medical chart review. This study protocol was approved by the ethical committee of each institution and informed consent from the patients was taken

### Statistical methods

Measurements of H2T and H2D were tested for association with OS, defined as the time from start of trastuzumab treatment to cancer-associated death or the end of the follow-up period. The log-transformed values of HER2 expression and HER2 homodimers were used for analyses. Deaths not attributed to breast cancer were considered as censored at the time of death. One patient was excluded from the analyses because she died merely 20 days after her first dose of trastuzumab, and the duration of treatment was considered insufficient to include the patient as "trastuzumab-treated."

Univariate analyses were performed using the Kaplan and Meier (KM) method, hazard function plots were estimated using the life-table method, and multivariate models were generated using Cox proportional hazards regression analysis. Cox proportional hazards models were fitted to examine the associations of H2T, H2D, or their ratio H2T/H2D with outcome, with adjustment for other potential prognostic factors. The adjustment factors included treatment group (trastuzumab-only or trastuzumab plus chemotherapy), estrogen and progesterone receptor status, and a composite of them, hormone receptor status, screening Herceptest score performed at the treating hospital, Herceptest score repeated at Monogram, Histoscore performed on repeat IHC at Monogram, tumor stage, and presence of metastases in the liver, lung, skin, bone, lymph nodes, brain, chest wall, or other viscera. Metastatic sites occurring in less than 10% of the total cohort were not considered as individual variables. The stepwise variable selection method was used to select the "best" models.

Statistical analyses were performed at Yokohama City University Medical Center using the statistical software packages SAS and R.

## Results and Discussion

The median duration of patient follow-up period was 18.2 months. The median duration of trastuzumab treatment was 14.6 months. A description of the clinical characteristics of the cohort is provided in Table 1. Of the 75 patients with HER2 test +2 or +3 assessed at the local hospitals, 25 had IHC 0 or +1 by the central pathological review. Nine patients had advanced disease, while 66 had recurrence. The number of pre-chemotherapy before trastuzumab treatment was also shown in Table 1. For the 66 patients with recurrence, the number of pre-chemotherapy received after recurrence was analyzed.

**Table 1:** Clinical characteristics of the cohort

Summary data			
**Characteristic**	**Cohort**		
Geographic origin	Japan		
Type of cohot	Clinic-based		
Centralized HER2 assessment	no		
			
Centralized clinical data collection	no		
Length of follow-up (months)	3.4-63.3		
			
Total Number of patients	75		

**Parameter**	**Number of patients (%)**		
HER2 status by IHC	75 (100)		
HER2 status by FISH	9(12.0)		

	**Total**	**Log_10 _HER2 expression • Median**	**Log_10 _HER2 expression • Median**

**HER2 test at the local hospital**			
2+	20 (26.7)	11 (28.9)	9 (24.3)
3+	55 (73.3)	27 (71.1)	28 (75.7)

**Centrally reviewed IHC**			
0	11 (14.7)	1 (2.6)	10 (27.0)
1+	14 (18.7)	2 (5.3)	12 (32.4)
2+	18 (24.0)	9 (23.7)	9 (24.3)
3+	32 (42.7)	26 (68.4)	6 (16.2)

**HER2 score on repeat testing**			
Mean (s.d.)	148 (101)	210 (71)	84 (87)
Min - Max	0 - 290	0 - 290	0 - 290

**Hormone receptor status**			
ER+ PR+	10 (13.3)	6 (15.8)	4 (10.8)
ER+ PR-	2 (2.7)	1 (2.6)	1 (2.7)
ER- PR+	3 (4.0)	0	3 (8.1)
ER- PR-	60 (80.0)	31 (81.6)	29 (78.4)
ER unknown, PR-	0	0	0
ER unknown, PR unknown	0	0	0

**Nodal status**			
Negative	21 (28.0)	14 (36.8)	7 (18.9)
1 to 3 positive nodes	23 (30.7)	9 (23.7)	14 (37.8)
4 to 10 positive nodes	8 (10.7)	4 (10.5)	4 (10.8)
> 10 positive nodes	17 (22.0)	7 (18.4)	10 (27.0)
Status missing	6 (8.0)	4 (10.5)	2 (5.4)

**Tumor size**			
< = 2 cm	9 (12.0)	5 (13.2)	4 (10.8)
>2 cm & < = 5 cm	37 (49.3)	19 (50.0)	18 (48.7)
>5 cm	17 (22.7)	6 (15.8)	11 (29.7)
Missing	12 (16.0)	8 (21.1)	4 (10.8)

**Prior adjuvant therapy**			
Adj HT	30 (40.0)	15 (39.5)	15 (40.5)
Adj CT	51 (68.0)	23 (60.5)	28 (75.7)
Adj HT only	5 (6.7)	2 (5.3)	3 (8.1)
Adj CT only	26 (34.7)	10 (26.3)	16 (43.2)
Adj HT & Adj CT	25 (33.3)	13 (34.2)	12 (32.4)
neither HT nor CT	19 (25.3)	13 (34.2)	6 (16.2)

Number of pre-chemotherapy before trastuzumab treatment*			
0	41 (54.7)	18 (47.4)	23 (62.2)
1	15 (20.0)	9 (23.7)	6 (16.2)
2 or 3	7 (9.3)	3 (7.9)	4 (10.8)
N.A.	12 (16.0)	8 (21.1)	4 (10.8)

**Tumor stage**			
Recurrence	66 (88.0)	32 (84.2)	34 (91.9)
Advanced	9 (12.0)	6 (15.8)	3 (8.1)

**Number of met sites**			
1 or 2	49 (65.3)	25 (65.8)	24 (64.9)
3 or 4	25 (33.3)	12 (31.6)	13 (35.1)
unknown	1 (1.3)	1 (2.6)	0

**Brain mets**			
yes	7 (9.3)	5 (13.2)	2 (5.4)
no	68 (90.7)	33 (86.8)	35 (94.6)

**Treatment**			
Herceptin + chemo	63 (84.0)	31 (81.6)	32 (86.5)
Herceptin only	12 (16.0)	7 (13.5)	5 (18.4)

* For the 66 patients with recurrence, the number of pre-chemotherapy received after recurrence was analyzed.

### Correlation of HERmark measurements with IHC

We have previously shown the relationship between HERmark measures of HER2 expression and IHC performed under standard conditions by a single pathologist in 170 FFPE breast tumors [16]. The results demonstrated a general correlation between the two assays, however the HERmark assay provided a more quantitative assessment of HER2 expression distributed over a 3-log dynamic range compared to the semi-quantitative readout of IHC (0-3+ scale) (see Additional file 1: Figure S1). We attempted to make the IHC measurement more quantitative by considering the histo-score (H score), where staining intensity is corrected for percent tumor involvement. The HERmark assay still demonstrated a greater ability to quantitate HER2 expression over a wider dynamic range.

**Figure 1:**
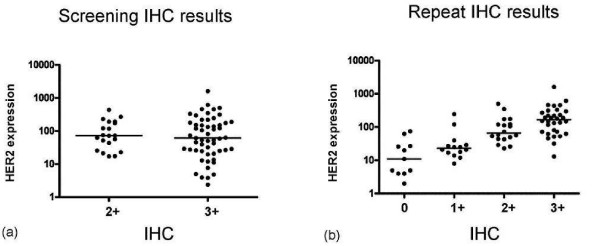
**Relationship between ICH results and HER mark measures of HER2**. (a) Screening IHC results correlated with HER2 expression. (b) Repeat IHC results, all patients (compare to Figure S1).

In this cohort, we initially compared H2T to the screening IHC calls provided by the treating hospitals. The distribution of H2T measurements ranged over a 3-log dynamic range, and there was no significant difference between the H2T distributions of those samples scored as 2+ or 3+ by the screening IHC assays (Figure 1a). We repeated the IHC assays on all specimens. The repeat assays were performed centrally and read by a single pathologist. Approximately one-third of the specimens were scored as 0 or 1+ upon repeat. Comparison of the repeat IHC results with the HER2 expression levels by HERmark yielded a stair-step correlation (Figure 1b) reminiscent of previous comparisons with centralized IHC results (see Additional file 1, Figure S1).

### Kaplan-Meier, hazard function plots, and multivariate Cox analyses

**Figure 2:**
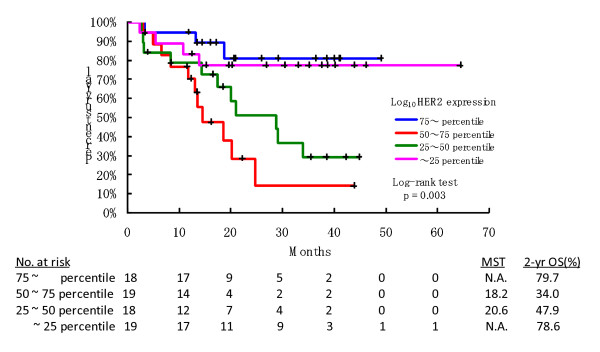
**Kaplan-Meier curve of OS by HER2 expression subgroup**.

**Figure 3:**
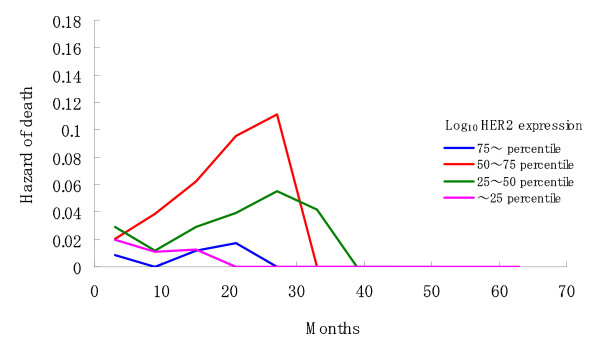
**Hazard function plots with six-month interval by HER2 expression subgroup**.

As a whole, two-year survival rate of the cohort was 60.8% (95% C.I., 48.4 - 73.2%). In the univariate analyses, patients were classified into four subgroups defined by the 25^th^, 50^th^, and 75^th ^percentiles for each of the three variables, H2T, H2D, and their ratio H2D/H2T, in order to examine the assumption of linearity for the continuous variables used in the subsequent Cox regression analysis. A significant difference in OS was found between the four subgroups with respect to H2T (Log rank test, p = 0.003; Figure 2), but not for the others (p = 0.371 and p = 0.573). The 25^th^, 50^th^, and 75^th ^percentiles for the log-transformed values of H2T were 1.41, 1.80, and 2.25, respectively. The patients' characteristics by H2T subgroup (< the median value of H2T: n = 37 v.s. • the median value of H2T: n = 37) is summarized in Table 1. Higher proportion of patients with greater H2T value than its median value had IHC 2+ or 3+ (p < 0.001) and higher HER2 score on repeat testing (p < 0.001). No significant difference in OS was found between the IHC subgroups (log-rank test: p = 0.610). Table 2 summarizes the other univariate analyses for OS. Figure 3 shows the hazard function plots estimated in the four H2T subgroups. The subgroups with the 25% highest and lowest H2T values had substantially lower risk of death than the middle two subgroups. These univariate analyses revealed a parabolic relationship between continuous measures of HER2 expression and risk of death, suggesting that the assumption of linearity for the HER2 expression measurements may be inappropriate in subsequent multivariate analyses. As shown in Table 3, this parabolic relationship was also confirmed by the Cox regression analysis using the categorized variable of H2T (compared with the reference subgroup having higher 50%-75% H2T, HR of the 25% highest = 0.25, p = 0.019, 95%CI: 0.08-0.77; HR of the 25%-50% = 0.79, p = 0.610, 95%CI: 0.32-1.97; HR of the 25% lowest = 0.12, p = 0.004, 95%CI: 0.03-0.51), which also shows that hormone receptor status (HR = 0.18, p = 0.014, 95%CI: 0.04-0.71) and brain metastases (HR = 0.07, p < 0.001, 95%CI: 0.02-0.24) were significant prognostic factors. Thus, the best multivariate model contained three variables, H2T, progesterone receptor status, and brain metastases.

**Table 2:** Univariate analysis for overall survival time.

Variable	Category	Hazard ratio	**95% C.I**.		P-value
Treatment group	trastuzumab/trastzumab+chemo	1.24	0.43	3.56	0.693
ER	+/-	0.81	0.28	2.33	0.694
PR	+/-	0.45	0.14	1.49	0.190
Hormone receptor	+/-	0.53	0.19	1.54	0.244
Tumor stage	Recurrence/advanced	3.30	0.45	24.4	0.242
No. of metastatic sites	1/2/3/4	0.54	0.37	0.79	0.002
Liver mets	No/Yes	0.74	0.34	1.59	0.435
Lung mets	No/Yes	0.83	0.40	1.72	0.615
Bone mets	No/Yes	0.42	0.20	0.88	0.021
Lymph node mets	No/Yes	1.02	0.49	2.14	0.959
Brain mets	No/Yes	0.18	0.07	0.46	<.0001

**Table 3:** Cox regression for survival time using the stepwise variable selection method.

Variable	Category	Hazard ratio	**95% C.I**.		P-value
Log_10_HER2 expression (1)	75 percentile~/50~ 75 percentile	0.25	0.08	0.77	0.019
Log_10_HER2 expression (2)	25~ 50 percentile/50~ 75 percentile	0.79	0.32	1.97	0.610
Log_10_HER2 expression (3)	~ 25 percentile/50~ 75 percentile	0.12	0.03	0.51	0.004
Hormone receptor status	+/-	0.18	0.04	0.71	0.014
Brain mets	No/Yes	0.07	0.02	0.24	<.0001

Dividing the cohort into high HER2-expressing (≥ the median value of H2T) and low HER2-expressing (< the median value of H2T) sub-groups and using the Cox regression analysis with the continuous H2T value in each of the two subgroups, we found that those patients with higher values for HER2 expression live longer than those with lower values for H2T in the high HER2-expressing group (HR = 0.06, p = 0.010, 95%CI: 0.01-0.51) (Table 4). In contrast, in the low HER2-expressing group, the opposite trend (those with lower H2T values are favored) was observed (HR = 16.0, p = 0.017, 95%CI: 1.64-155.9). These data suggest that there are two sub-populations of patients in this cohort that behave differently with respect to HER2 expression and OS.

**Table 4:** Cox regression analyses in two subgroups divided by the median HER2 expression value.

Cohort subset>	Variable	Hazard ratio	**95%C.I**.	P-value
Log_10_HER2 expression • 1.799*	Log10 HER2 expression	0.06	0.01 - 0.51	0.010
(n = 37)	Hormone receptor status	0.39	0.05 - 2.86	0.354
	Brain mets	0.14	0.02 - 0.87	0.034

Log10HER2 expression < 1.799*	Log10 HER2 expression	16.0	1.64 - 155.9	0.017
(n = 37)	Hormone receptor status	0.20	0.03 - 1.57	0.125
	Brain mets	0.04	0.01 - 0.28	0.001

* Median (50 percentile) value of Log_10_HER2 expression measurements

Trastuzumab is a remarkably effective drug for patients with breast cancer who over-express HER2, or have evidence of HER2 gene amplification. However, not all patients with MBC respond to trastuzumab, perhaps because not all patients who receive the drug are truly over-expressing HER2, or perhaps because some proportion of those that do over-express HER2 also signal through other pathways that are not antagonized effectively by trastuzumab. Such patients may be expected to experience fleeting responses when treated with trastuzumab, or even to show no evidence of clinical response at all. As the ability to accurately assess such escape pathways gradually evolves, we should be able to identify the mechanisms of resistance to trastuzumab, so that effective strategies for combination therapy can be developed and tested in prospective clinical trials. That process will take time, but perhaps a first step could be to improve our ability to accurately quantitate HER2 protein expression and try to gain a better understanding of how differences in expression levels among individual breast tumors affect the natural history of HER2-positive breast cancer and, more importantly, response to HER2-targeted agents like trastuzumab.

Recently presented data at the 2007 ASCO annual meeting have raised questions about the accuracy of current testing methods [12-14]. In particular, a re-analysis of the central testing data from the NSABP B-31 trial demonstrated that nearly 10% of the trastuzumab-treated population in the trial were IHC < 3+, FISH-negative on repeat testing, but benefited from trastuzumab therapy just as the IHC 3+ and FISH-positive groups did [14]. Perhaps this observation reflects fundamental differences between the pathophysiology of breast cancer in the adjuvant setting compared with the metastatic setting, or perhaps we need better tests for HER2. Whatever the case, there appears to be a genuine need to re-examine the issue of patient selection for trastuzumab, with an eye toward new technologies and new approaches that have the potential not only to improve current standards, but also to begin identifying alternative signaling complexes that can be targeted by combination therapy regimens including trastuzumab.

In this study, we describe the relationship between quantitative measures of HER2 expression or HER2 homodimers and overall survival in a cohort of patients with MBC who were treated with trastuzumab. The assay system used to make these measurements is CLIA validated for accuracy and reproducibility, and by virtue of its ability to quantitate proteins and protein dimers, provides a more granular view of the HER2 landscape than either IHC or FISH [16]. The assay has previously been correlated with clinical outcomes following trastuzumab therapy in a cohort of MBC patients who were very carefully selected for treatment using FISH at the Jules Bordet Institute in Belgium [15].

The current study represents the second clinical experience with the HERmark assay, involving a group of patients who were selected for trastuzumab treatment primarily by IHC (88%) performed in the course of routine clinical care. The initial KM analyses and hazard function plots of the entire cohort suggested that two distinct populations were present, and the observed relationships between HER2 expression and OS for the two sub-groups were going in opposite directions. Using a cutoff of the median value of log_10 _HER2 expression = 1.80, we then re-analyzed the patient sub-group that had HER2 expression values greater than the cutoff to see whether this sub-population showed any evidence that higher levels of HER2 expression yielded longer OS on trastuzumab. Data from the Bordet cohort described previously had shown such an association, and we wanted to see if we could confirm this relationship in the current study. Because the Bordet cohort was so stringently selected for HER2 over-expression or gene amplification, patients with low HER2-expression, such as those that represented approximately one-third of the current cohort, may have been excluded. Given the differences in the methodology used to select patients in the two cohorts (90% FISH in Bordet, 88% IHC in the current study), as well as the stringency applied (expanded access cohort with mandatory central confirmation of HER2 status in Bordet, routine clinical care in the current study), it is perhaps not so surprising that we might observe this difference. These data appear to confirm the relationships that were seen in the Bordet cohort and also suggest that, within a population of patients that are identified as HER2-positive by the commonly used methods currently available to clinical oncologists, precise quantitation of HER2 expression can define multiple sub-populations of patients that experience different clinical outcomes in response to trastuzumab treatment.

These data suggest that patients can be characterized according to the degree of HER2 protein expression that their tumors exhibit, and that quantitative expression levels matter clinically. It is important to note that these data do not address the question of whether some patients treated with trastuzumab may not benefit clinically. Because we tested only patients who were treated with trastuzumab, the finding that some did not benefit as much as others does not equate to some did not benefit at all. Prospective studies or retrospective analyses of prospectively designed clinical trials where HER2-positive patients were randomized to receive trastuzumab or not will need to be tested in order to answer that question. Nonetheless, data from the current study demonstrate that trastuzumab-treated patients with low HER2 expression levels appear to behave differently with respect to OS than those with high HER2 expression levels. Whether this subset of patients behaves differently because they exhibit normal rather than elevated but low levels of HER2 is a matter for future study. In addition, we should have carried out the analyses to examine the relationship between HER2 expression levels and OS in patients with IHC 2+ or 3+. We could not obtain statistically reliable results, however, because the number of patients included in the subgroup was small (n = 50) and, in particular, the number of patients with brain metastases was only 5. This is also a matter for future study.

What do these data imply about the impact of trastuzumab on HER2-positive tumors that express different levels of the target? Previous data have shown that the amount of HER2 protein expressed correlates with disease progression in MBC patients not treated with trastuzumab [3]. Data from the current study are restricted to patients that have been treated with trastuzumab, but nonetheless support the general conclusion that the amount of HER2 expression matters clinically. One potential explanation for such a finding is that the amount of HER2 expression is a marker for the degree of HER2 dependency that exists in the tumor. Tumors that massively over-express HER2 may be "addicted" to signaling through HER2, potentially explaining their association with rapid progression in the absence of trastuzumab as well as enhanced susceptibility to antagonism by trastuzumab. Tumors which express lower levels of HER2 may be less vulnerable to trastuzumab because they are less dependent on HER2 alone, but rather derive at least some of their proliferative drive from protein-protein associations (HER1:HER2 or HER2:HER3 heterodimers) or variants (p95/HER2) that are not as susceptible to antagonism by trastuzumab [17-23]. Other explanations involving mutations in downstream components (PTEN) that affect PI3-kinase signaling activity as well as involvement of alternative signaling pathways (eg. IGF-1R) are equally plausible [24,25]. Alternatively, the association between higher levels of HER2 expression and better outcomes on trastuzumab may reflect enhanced targeting of HER2 over-expressing cells by immune mechanisms triggered by trastuzumab binding, like antibody dependent cellular cytotoxicity (ADCC). Perhaps tumors at the lowest end of the HER2-expression distribution, that appear to have favored outcomes on trastuzumab, are simply expressing normal levels of HER2, and thus have a fundamentally different prognosis than their counterparts expressing significantly higher levels of HER2. These questions, as well as the general finding that, among tumors already classified as HER2 over-expressing or gene-amplified by standard assays, precise assessments of HER2 expression levels correlate with clinical outcomes following trastuzumab treatment, demand further scrutiny in larger, well-controlled trials of patients with metastatic breast cancer.

Although the technology enabling quantitative assessments of potential escape pathways for trastuzumab is still evolving, the opportunity to begin to sub-divide populations of patients into their constituent groups based on precise measurements of HER2 and its family members holds tremendous promise at a time when numerous targeted drugs are becoming available for inclusion in combination therapy regimens for patients with breast cancer. It is our hope that observations derived from the current study represent an early step in that direction.

## Conclusion

It is concluded that the quantitive HER2 measurement by Hermark assay may be useful for identifying a more relevant target population to trastuzumab treatment in patients with MBC. It is warranted to study the significance of the quantitive HER2 measurement in the adjuvant situation with anti-HER2 therapy for primary breast cancer patients.

## Competing interests

JS, WH, JW, XJ, YT, CP, and MB are employees and stockholders in Monogram Biosciences.

## Authors' contributions

MT carried out study design, clinical study coordination, hypothesis generation, supervision of data analyses, and data interpretation. Carried out JS data quality assurance, data analysis, and data interpretation. WH participated in central IHC re-tests, oversight of HERmark assay performance, data interpretation. SS participated in study design, data management, clinical trial. JW carried out HERmark assay design and supervision of HERmark data generation. XJ and YT carried out HERmark assay performance and quality assurance. SO, SN, HI, NM, and KA participated in clinical trial. SM carried out biostatistical analysis, supervision of data analysis, and data interpretation. CP conceived of assay design and supervised all HERmark assay development, data interpretation. MB carried out study design, hypothesis generation, supervision of data analyses, data interpretation. MT and MB have manuscript authorship. All authors read and approved the final manuscript.

## Pre-publication history

The pre-publication history for this paper can be accessed here:

http://www.biomedcentral.com/1471-2407/10/56/prepub

## Supplementary Material

Additional file 1 **Figure S1. Correlation of HER2 expression Vera Tag and IHC in 170 breast tumors (FFPE)**. The correlation between the Vera Tag measure of HER2 expression and IHC in 170 FFPE breast tumor specimens. This is a reproduction of figure 6a from Shi, et al. (footnote 2) provided here for convenience. Click here for file
