# Supplementary figures and images for: Differential survival following trastuzumab treatment based on quantitative HER2 expression and HER2 homodimers in a clinic-based cohort of patients with metastatic breast cancer
Source: BMC Cancer. 2010 Feb 23;10:56. doi: 10.1186/1471-2407-10-56 (PMC2837013; doi:10.1186/1471-2407-10-56)

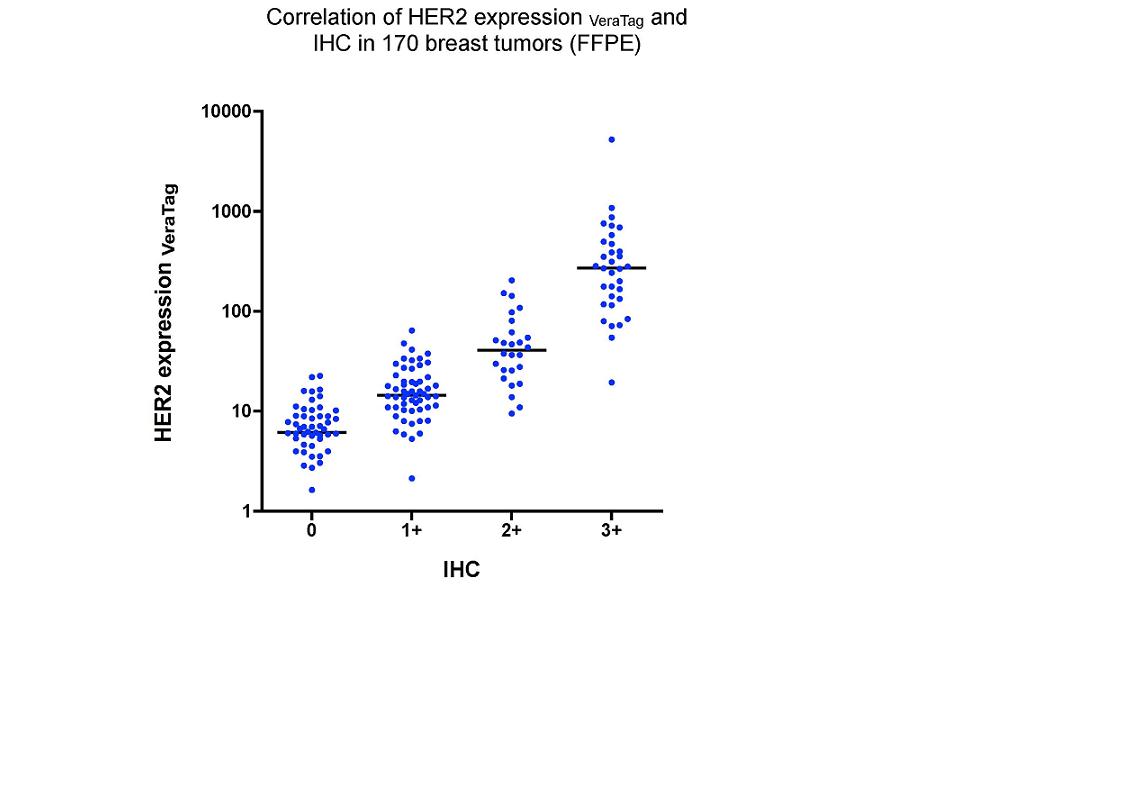

Supplement: Additional file 1 — Figure S1. Correlation of HER2 expression Vera Tag and IHC in 170 breast tumors (FFPE). The correlation between the Vera Tag measure of HER2 expression and IHC in 170 FFPE breast tumor specimens. This is a reproduction of figure 6a from Shi, et al. (footnote 2) provided here for convenience. [file 1471-2407-10-56-S1.DOC]
